# Mathematical modeling of rare earth element separation in electrodialysis with adjacent anion exchange membranes and ethylenediaminetetraacetic acid as chelating agent

**DOI:** 10.1038/s41598-024-62885-4

**Published:** 2024-05-28

**Authors:** Lingyang Ding, Gisele Azimi

**Affiliations:** Laboratory for Strategic Materials, Department of Chemical Engineering and Applied Chemistry, University of Toronto, 200 College Street, Toronto, ON M5S 3E5 Canada

**Keywords:** Rare earth elements, Separation, Ethylenediaminetetraacetic acid, Electrodialysis, Mathematical modeling, Extended Nernst–Planck equation, Chemical engineering, Process chemistry

## Abstract

This research delves into the effective use of electrodialysis for the separation of rare earth elements (REEs), specifically separating dysprosium (Dy) from praseodymium (Pr) and neodymium (Nd). A robust mathematical model based on the extended Nernst-Planck equation is introduced, simulating the process within a configuration that includes two adjacent anion exchange membranes. The model integrates aspects such as feed equilibrium, ion exchange within the membrane, and overall ion flux. Validation of the model's predictability was conducted through Chi-squared tests and root mean square error (RMSE) calculations, affirming its capability to accurately predict ion concentrations across different compartments. The study examines essential parameters such as applied voltage, rinse solution concentration, and feed concentration, assessing their impacts on separation performance and energy efficiency. Results indicate that higher voltages above 8 V, while speeding up separation, detrimentally impact energy use. It also highlights a critical balance in rinse solution concentration; lower concentrations below 0.05 mol/L enhance energy efficiency but may undercut separation efficacy due to early depletion. A linear correlation between the necessary rinse concentration and feed concentration was established, with higher feed concentrations demonstrating reduced specific energy consumption, thus enhancing overall efficiency. However, challenges remain in current efficiency due to the independent migration of SO_4_^2−^ ions in this specific setup. The findings advocate exploring alternative configurations, like alternating cation and anion exchange membranes, to optimize both environmental and economic aspects of REE separation. This study provides valuable insights and recommendations for refining electrodialysis systems in REE processing, contributing to sustainable and cost-effective electrodialysis systems.

## Introduction

Rare earth elements (REEs), known for their unique physical and chemical properties, are crucial in various advanced technological domains, including magnetics, electronics, optics, ceramics, catalysis, hydrogen storage, and batteries. Their importance in these fields, particularly in clean energy and defense applications, has led to their recognition as critical materials in several countries. This classification underscores the vital role REEs play in supporting and advancing modern technology and infrastructure^1^.

The REE industry is confronted with the complex task of separating these elements, a challenge stemming from their similar physicochemical properties. Traditional separation methods include precipitation, ion exchange, and solvent extraction. The commonly used precipitation method employs oxalic acid to create mixed REE oxalates, but this approach is not only time-consuming, but it also struggles to achieve high purity levels of 99.9% in the final REE products. Ion exchange, while capable of refining all REEs and adaptable to various raw material compositions, is hampered by the low concentration of REEs in solutions, leading to prolonged batch processes that can last up to a month, thus resulting in low productivity and increased operational costs. Alternatively, the solvent extraction method, prevalent in the REE industry, offers a continuous process capable of handling higher concentrations, which notably reduces residence time. However, this method faces difficulties in achieving high-purity products and maintaining stable operations, especially when separating REEs with a separation factor close to 2, even with 40–50 extraction stages^2^. Given these advantages and limitations of conventional separation and purification techniques, it becomes evident that there is an urgent need for new and innovative separation technologies. These advanced methods should be capable of efficiently producing high-purity REEs, particularly in a global context where the demand for these elements is steadily increasing.

In recent times, the electrodialysis method has been explored for the separation of REEs, offering a range of benefits. Its simplicity and ability to operate continuously make it well-suited for multi-stage processes. Electrodialysis is not new; it has been widely applied in various industrial applications such as brackish water desalination, purification of organic solutions, and treatment of municipal or industrial effluents^3–5^. Additionally, electrodialysis has been utilized in separating lithium from cobalt^6^, as well as in separating lithium, cobalt, nickel, and manganese from the NMC (nickel manganese cobalt oxide) cathode material during the recycling of lithium-ion batteries^7^.

Historically, there has been a scarcity of research on using electrodialysis for separating REEs^2,8^. Recently, however, interest in this area has surged, and several research groups, including ours, have started to explore this application^9–12^. One of the challenges in electrodialysis for REE separation is the low selectivity of ion exchange membranes for ions of equal valence, making the separation of REEs from mixed solutions difficult. To improve selectivity, chelating agents, such as aminocarboxylic acids, are employed. These agents form stable chelates with REE ions, and the strength of this bond is quantified by an absolute stability constant (K_ABS_), indicating the affinity of the chelating agent for a specific REE. In electrodialysis, this stability constant is crucial in determining the balance between the chelate and its ionized forms in the solution. Generally, light REEs exhibit lower stability constants compared with heavy REEs, leading to a competition among positively charged ions, including protons and REEs, for chelation sites in the presence of multiple metal ions. Typically, the chelating agent will first react with the ion that forms the most stable chelate (the highest stability constant) and, if there is excess agent, it will then react with ions having lower stability constants. However, the separation of REEs poses a unique challenge due to their closely similar stability constants and minimal differences between them. This results in a simultaneous competition for chelation, causing various metal-chelate complexes to form concurrently in the solution. This complexity underscores the need for advanced research and innovative approaches in the electrodialysis process for effective REE separation^13^. The REE-chelate solution can be refined by modifying the pH and the quantity of chelating agents prior to advancing to the subsequent electrodialysis stage (cascade design). Once the purity of the REE-chelate solution meets the desired standards, a precipitating agent like ammonium bicarbonate is utilized to convert REEs into solid rare earth carbonate. Complete precipitation of REEs with ammonium bicarbonate occurs at a pH between 6 and 7.5. This solid rare earth carbonate is then calcined to produce the final product, rare earth oxide^14^.

The ion transport mathematical model is crucial in understanding electrodialysis process, offering a detailed view of electrotransport within the electrodialysis cell. This model is instrumental in shedding light on ion transport mechanisms and predicting the performance of electrodialysis. It encompasses various geometric scales, each focusing on a distinct aspect of the process: (1) the Membrane scale which zeroes in on the membrane's behavior and properties. It explores the characteristics of the membrane material, including aspects like ion selectivity and permeability, crucial for understanding how ions move through the membrane. (2) The Three-layer system which examines the interaction between the membrane and the two diffusion layers adjacent to it. It focuses on the ion transport mechanisms within this trio, comprising the membrane and the solution layers on either side, offering insights into how ions traverse these layers. (3) Hydraulic flow coupling which considers the dynamics of hydraulic flow within the electrodialysis cell. It encompasses fluid dynamics and transport phenomena in both two-dimensional and three-dimensional setups, providing a more comprehensive view of how ions are distributed spatially within the cell. Together, these scales form an intricate framework for understanding ion transport in electrodialysis. By delving into each scale, researchers and engineers can gain valuable insights into how the electrodialysis process functions, predict its efficacy, and fine-tune its operations for a variety of applications^15^. This multi-scale approach is key to advancing the efficiency and application of electrodialysis in various industrial contexts.

**Figure 1:**
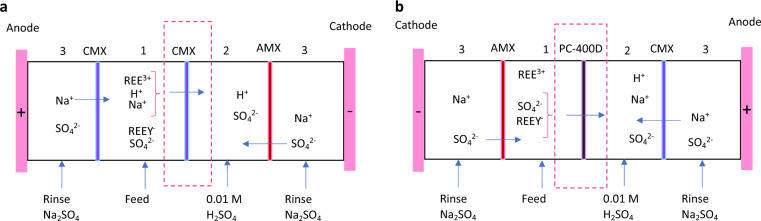
(**a**) A schematic diagram of the electrodialysis cell with one cation exchange membrane separating the feed and concentrate compartments allowing for passage of cationic species. (**b**) A schematic diagram of the electrodialysis cell with one anion exchange membrane (PC-400D) separating the feed and concentrate compartments allowing for passage of anionic species. *AMX* anion exchange membrane, *CMX* cation exchange membrane, *PC-400D* anion exchange membrane.

Electrodialysis for separating REEs can be implemented using two different cell and membrane setups, as depicted in Fig. 1a,b. In these systems, ethylenediaminetetraacetic acid (EDTA, C_10_H_16_N_2_O_8_) is employed as the chelating agent, forming an anionic complex with REEs (notated as REEY^−^). In the setup illustrated in Fig. 1a, a cation exchange membrane (CMX) is utilized to divide the feed (1) and concentrate (2) compartments. This CMX permits the passage of all cations, including REE(III)^3+^, H^+^, and Na^+^, from the feed to the concentrate compartment. Consequently, lighter REE(III) ions, which remain unchelated, are transported to the concentrate compartment, while heavier REE(III) ions, chelated with EDTA, stay in the feed compartment.

Conversely, in Fig. 1b, an anion exchange membrane (PC-400D), which allows large anions to pass, is employed. This membrane enables the migration of REEY^−^ and SO_4_^2−^ anions from the feed to the concentrate compartment, resulting in the concentration of heavier, chelated REE(III) ions in the concentrate compartment, while lighter, free REE(III) ions remain in the feed compartment. The setup in Fig. 1a is more commonly used in previous studies on REE separation via electrodialysis. In a recent study, our group developed a phenomenological model for the setup using a CMX membrane^12^. This model encompasses the transport of all cations through the membrane.

In another research effort, we explored the separation of dysprosium (Dy), a heavy REE, from a mixture of neodymium (Nd) and praseodymium (Pr), which are lighter REEs, using EDTA as the chelating agent^11^. This study employed the setup shown in Fig. 1b, where anions migrate through the anion exchange membrane (PC-400D). No prior studies have developed a mathematical model for REE-chelate migration through the anion exchange membranes.

Developing such a model would be crucial in determining optimal operational parameters in the electrodialysis cell presented in Fig. 1b, including chelating agent quantities, pH levels, applied voltage, initial feed and rinse solution concentrations, and their impact on product purity, yield, separation efficiency, electrodialysis duration, and specific energy consumption. The lack of a model for anion exchange membranes in the REE separation underscores the opportunity for significant advancements in refining electrodialysis processes for REE separation.

In this study, we employed Class 1 models (Transport in Membrane) to formulate a mathematical model for separating Dy from Nd and Pr. This model is based on the Nernst-Planck equation, focusing on chelation-assisted electrodialysis. A key element in this process is the use of EDTA in the form of Na_2_EDTA as the chelating agent. The developed model features several adjustable inputs, including the initial REE(III) concentration, the quantity of Na_2_EDTA, the feed pH, the applied voltage, the concentration of the rinsing solution (Na_2_SO_4_), and the duration of the process. The outputs of the model include the concentrations of REE(III) ions in the feed and concentrate solutions, the concentration of Na^+^ and SO_4_^2−^ in the rinse solution, feed solution, and concentrate solution, and the fluxes of the REEY^−^ ions and SO_4_^2−^ through PC-400D anion exchange membrane.

The experimental study^11^ explored a range of pH and EDTA to Dy molar ratios, concluding that a pH of 4 and an EDTA/Dy molar ratio of 1 provided optimal separation. Using the model, we examined how other parameters including applied voltage, rinse solution concentration, and feed concentration affect separation performance and energy efficiency. Key performance indicators (KPIs) such as the purity (%) of Dy, the yield (%) of Dy, and the separation factor were calculated. Additionally, specific energy consumptions were evaluated to gauge energy efficiency. To validate the model's predictive capacity, we compared it with experimental results of a pervious study^11^ using a Chi-squared (${\chi }^{2}$) test to assess the predicted versus experimental values of REE concentration in both feed and concentrate compartments. The root mean square error was also calculated to determine the quality of the model's predictions. The detailed experimental procedures and results are reported in our previous study^11^.

Developing a new model for a different cell configuration in electrodialysis, while building on a previous model^12^ that already offers significant advantages, can bring additional, specific benefits including tailored optimization for specific configurations, enhanced understanding of different configurations, comparative analysis and benchmarking, and furthering academic and scientific knowledge. Developing a new model for a different cell setup in electrodialysis can provide specific advantages in terms of optimization, understanding, and application, tailored to the unique characteristics of that particular configuration, potentially opening up new avenues of research.

## Results and discussion

### Experimental verification of the model

To assess the accuracy of the developed model, we compared its predictions to experimental results from three electrodialysis experiments conducted at different voltages: 10 V, 12 V, and 14 V. Other experimental conditions remained consistent across these tests, including a feed solution pH of 4, an EDTA/Dy molar ratio of 1, and a 0.05 mol/L Na_2_SO_4_ rinse solution, and feed containing 1 mmol/L Pr(III), Nd(III), and Dy(III) (140, 144, 162 mg/L, respectively) at 25 °C as detailed in our previous publication^11^.

In the comparison, the focus is put on the concentrations of Pr and Nd in the feed compartment and Dy in the concentrate compartment, as these are the primary REEs in their respective compartments. To evaluate the model's fitness, a Chi-squared (${\chi }^{2}$) test was conducted. In each experiment, 14 data points were collected, resulting in a degree of freedom of 13 for both Pr and Nd. For Dy, however, the degree of freedom was 11 due to the requirement that values for the ${\chi }^{2}$ test must be greater than 5, and the initial two values for Dy did not meet this criterion. Despite this, the ${\chi }^{2}$ test was conducted, and the corresponding confidence level was calculated. Additionally, the RMSE (Root-Mean-Square Error) was calculated to estimate the average error between the model predictions and experimental values.

**Figure 2:**
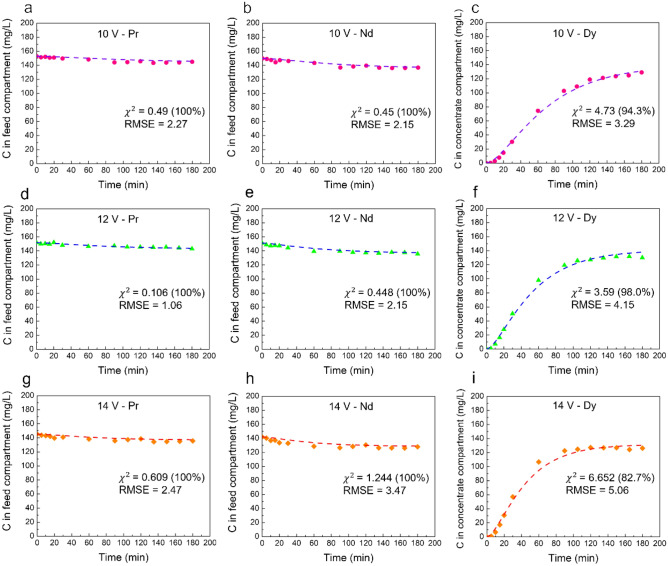
Comparison between the experimental data and model prediction. (**a**–**c**) The concentration of Pr and Nd in feed compartment and Dy in concentrate compartment at 10 V applied potential. (**d**–**f**) The concentration of Pr and Nd in feed compartment and Dy in concentrate compartment at 12 V applied potential. (**g**–**i**) The concentration of Pr and Nd in feed compartment and Dy in concentrate compartment at 14 V applied potential. Other conditions: feed solution pH of 4, an EDTA/Dy molar ratio of 1, and a 0.05 mol/L Na_2_SO_4_ rinse solution, and feed containing 140, 144, 162 mg/L of Pr(III), Nd(III), and Dy(III) at 25 °C, respectively.

The comparison, visualized in Fig. 2, shows the ${\chi }^{2}$, confidence level, and RMSE values. The time lag constant value c in the model was determined to be 1000, 600, and 600 for the experiments at 10 V, 12 V, and 14 V, respectively. This indicates that lower voltage experiments have a larger c value, reflecting a longer initial ion migration delay through the membrane due to the lower driving force. The similarity in c values for 12 V and 14 V suggests that increasing the voltage beyond 12 V does not significantly reduce this initial time lag.

The model predicts the concentrations of praseodymium (Pr) and neodymium (Nd) with high accuracy, characterized by low χ^2^ values and a 100% confidence level. However, the confidence in the predictions for dysprosium (Dy) is not as robust, particularly at higher voltages such as 14 V, where it only achieves a confidence level of 82.7%. Since the χ^2^ distribution is continuous, it encompasses an infinite number of confidence levels corresponding to various points along the χ^2^ distribution curve. However, standard χ^2^ tables typically only include the most common confidence levels and their associated critical χ^2^ values for different degrees of freedom. In this analysis, given the varying predictability of the model for the concentrations of Pr, Nd, and Dy, we find it useful to report the confidence levels based on their specific χ^2^ values and degrees of freedom. This approach allows readers to clearly see that the model's accuracy in predicting Dy concentration is not as robust as it is for Pr and Nd. The RMSE values are generally smaller for the 10 V and 12 V tests compared with the 14 V test, indicating better prediction accuracy at lower voltages. Overall, the errors are deemed acceptable given the large concentration values involved, confirming the model's effectiveness and reliability in predicting ion concentrations in the electrodialysis process.

### Effect of applied voltage on separation process

In electrodialysis, voltage plays a crucial role in both time efficiency and energy efficiency of the process. According to the Nernst–Planck equation, voltage acts as the primary driving force for ion migration. Its magnitude has a direct impact on the ion migration flux and, consequently, on the speed of the separation process. However, while time efficiency is important, energy efficiency is often of greater concern, especially when considering the comparative performance of electrodialysis against traditional solvent extraction methods for separating REEs.

Previous studies have indicated that one potential disadvantage of electrodialysis compared with solvent extraction in REE separation is its energy efficiency^11^. Therefore, understanding and improving the energy efficiency of electrodialysis is crucial for making it a competitive technology in the REE separation industry.

**Figure 3:**
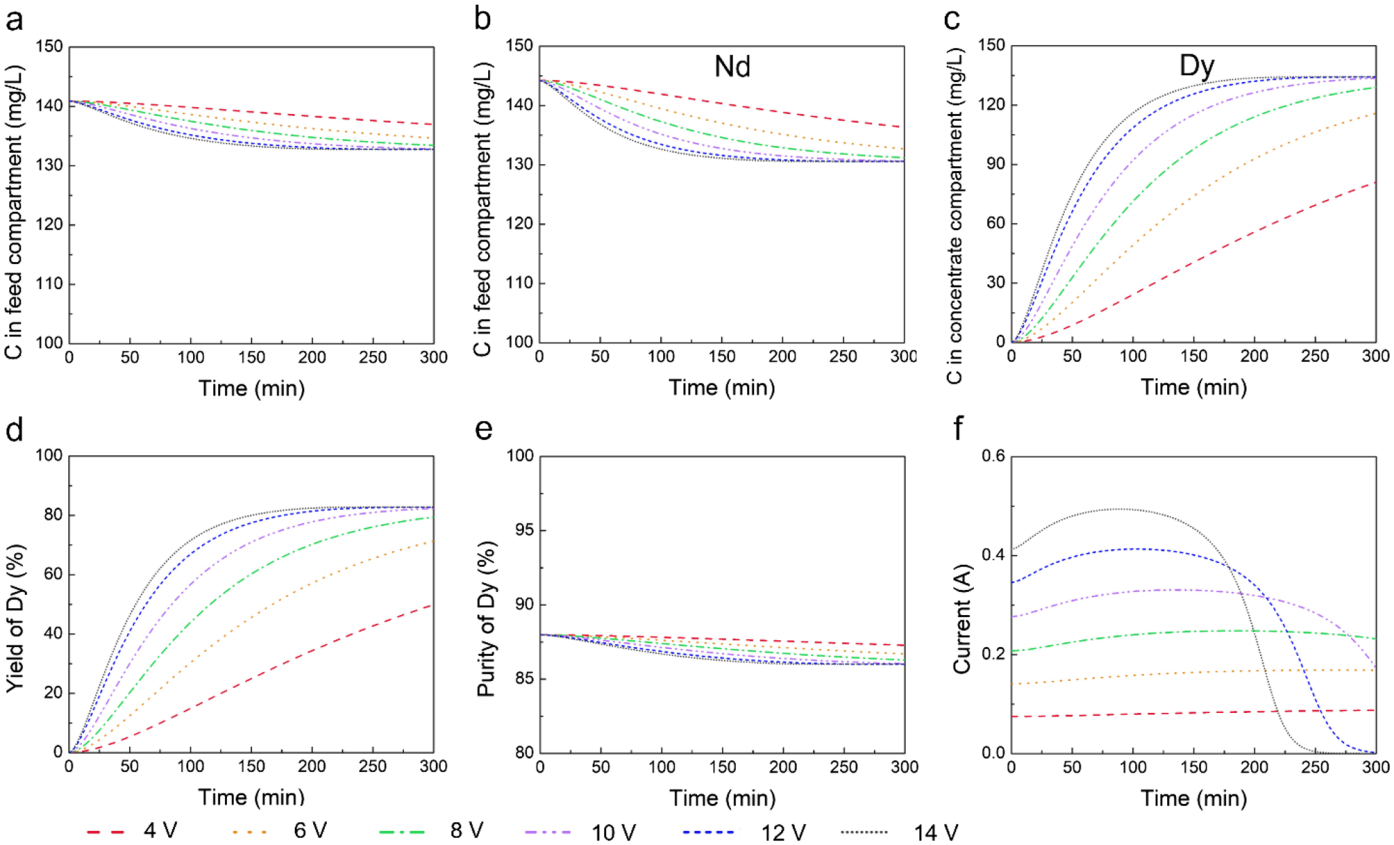
Separation performance and current during the process at different applied voltages. (**a**–**c**) Concentration of Pr(III) and Nd(III) in feed compartment and Dy(III) in concentrate compartment. (**d**) Yield of Dy. (**e**) Purity of Dy. (**f**) Current. Other conditions: feed solution pH of 4, an EDTA/Dy molar ratio of 1, and a 0.05 mol/L Na_2_SO_4_ rinse solution, and feed containing 1 mmol/L Pr(III), Nd(III), and Dy(III) at 25 °C.

We simulated electrodialysis processes at six different voltages: 4, 6, 8, 10, 12, and 14 V. The simulations were conducted with an initial REE concentration of 1 mmol/L for Pr(III), Nd(III), and Dy(III) at 25 °C, an EDTA/Dy molar ratio of 1, and a feed pH of 4. Figure 3a–c illustrate the concentrations of Pr(III) and Nd(III) in the feed compartment and Dy(III) in the concentrate compartment, respectively. The dynamic changes in concentrations during the separation process reveal that higher voltages lead to faster ion migration. For instance, at 10 V and 12 V, the separation process approaches completion before 150 min, while at lower voltages like 4 V and 6 V, the process has not yet reached a plateau even after 300 min. This observation highlights the influence of voltage on the speed of ion migration and separation, which is a critical factor in evaluating the overall efficiency of the electrodialysis process. Optimizing voltage not only improves the time efficiency of the separation but it is also key to enhancing the energy efficiency, making electrodialysis a more viable option for REE separation.

Figure 3d,e display the yield and purity of Dy, respectively. Notably, there is an observable trade-off where an increase in yield results in a decrease in purity. To attain both high yield and high purity, a cascading design could be an effective strategy in industrial applications. Interestingly, at the end of the experiments, across all voltages, the same Dy yield (82%), purity (86%), and separation factor (56) were achieved. This uniformity suggests that voltage does not significantly influence the overall separation performance.

In electrodialysis, electric energy is expended primarily to facilitate three key processes: the activation of hydrogen and oxygen evolution reactions at the electrodes, the movement of ions through the membranes, and unavoidable thermal losses. Among these, the ion migration through the membranes—referred to as ohmic consumption—dominates due to its significant contribution to the overall potential drop across the system. In our study, we focus on the total energy consumption, which is determined by monitoring the current and voltage throughout the electrodialysis process. This total energy usage is then normalized to the quantity of Dy separated, allowing us to compute the specific energy consumption (SEC). The SEC is utilized as a metric to gauge the energy efficiency of the process based on Dy recovery. This approach not only underscores the efficiency of the system but also highlights the effectiveness of our method in enhancing the sustainability of rare earth element separation. Figure 3f presents the current during the process. Notably, the maximum current at 14 V was close to 0.5 A, which is still in the normal range for electrodialysis applications. To compare energy efficiency across different experiments, we calculated specific energy consumption when all experiments reached 82% Dy yield. These measurements, alongside time requirements and separation performance, are summarized in Table 1.

**Table 1:** Electrodialysis time, Dy yield, purity, separation factor, specific energy consumption, and specific energy contributed by overpotential at different applied voltages.

Voltage (V)	Electrodialysis time (min)	Dy yield (%)	Dy purity (%)	Separation factor	SEC (kWh/mol)	SEC contributed by overpotential (%)
4	1041	82	86	56	14	15.0
6	564	82	86	56	21	11.3
8	387	82	86	56	29	9.5
10	284	82	86	56	36	7.6
12	217	82	86	56	41	6.3
14	184	82	86	56	48	5.7

Our findings indicate that higher voltages can reduce separation time but do not enhance separation performance. However, voltage markedly influences specific energy consumption, which dramatically decreases from 48 kWh/mol at 14 V to 14 kWh/mol at 4 V. This significant observation suggests that operating electrodialysis at lower voltages can lead to better energy efficiency. The contribution of overpotential to the SEC is detailed in Table 1. As the voltage decreases, a relatively larger proportion of energy is consumed by electrode reactions, which may appear less energy-efficient at lower voltages. However, for industrial applications, it is the overall SEC that impacts economic feasibility. Therefore, the focus should remain on optimizing the overall SEC, as this is the critical factor determining the economic viability of the process.

### Effect of rinse solution concentration

**Figure 4:**
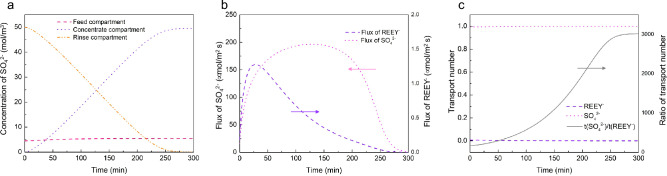
(**a**) Concentration of SO_4_^2−^ in each compartment. (**b**) Flux of SO_4_^2−^ and REEY^−^. (**c**) Transport number of SO_4_^2−^ and REEY^−^ and their ratio. Other conditions: feed solution pH of 4, an EDTA/Dy molar ratio of 1, applied voltage of 12 V, and feed containing 1 mmol/L Pr(III), Nd(III), and Dy(III) at 25 °C.

The rinse solution in electrodialysis plays a crucial role in neutralizing the charge in the feed and concentrate compartments and significantly influences the current of the process. Consequently, its concentration impacts both the separation performance and the energy efficiency of the system. Figure 4a illustrates the changes in SO_4_^2−^ ions concentration across different compartments during the process. As SO_4_^2−^ from the rinse solution continuously migrates to the feed compartment and accumulates in the concentrate compartment, its concentration decreases in the rinse solution and increases in the concentrate compartment. In the feed compartment, the concentration of SO_4_^2−^ remains relatively stable since these ions do not stay there permanently and some are required to neutralize REE(III)^3+^ ions.

Figure 4b displays the flux of SO_4_^2−^ compared with the total flux of REEY^−^ chelates. It is evident that the flux of SO_4_^2−^ is significantly higher than that of REEY^−^, which accounts for the low current efficiency observed in this cell configuration. Figure 4c displays the transport numbers for the ions REEY^−^ and SO_4_^2−^, along with the ratio of their transport numbers. The transport number for SO_4_^2−^ approaches 1, whereas the transport number for REEY^−^ is near zero, indicating low current efficiency in the current cell configuration. As the process continues, the concentration of REEY^−^ in the feed compartment decreases, while the concentration of SO_4_^2−^ remains relatively stable, leading to an increasing disparity in their transport numbers. As depicted in Fig. 4c, the ratio of their transport numbers escalates from 171 at the start to 3020 after 300 min, demonstrating a decrease in energy efficiency with longer operation times. The relatively high concentration of SO_4_^2−^ (0.05 mol/L) compared with REEY^−^ (1 mmol/L), along with its larger charge value, makes SO_4_^2−^ ions more competitive in migrating through the electrodialysis system, and the competition is more pronounced as time increases. This higher migration rate of SO_4_^2−^ ions is not desirable as it leads to lower energy efficiency.

One potential approach to enhance energy efficiency is to reduce the concentration of the rinse solution. However, this adjustment can impact the separation performance, creating a trade-off. This trade-off is further explored in subsequent simulations that investigate varying concentrations of the rinse solution. By examining these scenarios, we aim to find a balance that optimizes both the energy efficiency and the effectiveness of the separation process in electrodialysis.

In this study, we explored the effects of using lower concentrations of Na_2_SO_4_ (0.02, 0.03, and 0.04 mol/L) in the electrodialysis process, in comparison with 0.05 mol/L. Other experimental conditions were kept constant, including a 1 mmol/L Pr(III), Nd(III), and Dy(III) feed concentration, an EDTA/Dy molar ratio of 1, a feed solution pH of 4, and an applied voltage of 4 V at 25 °C.

**Figure 5:**
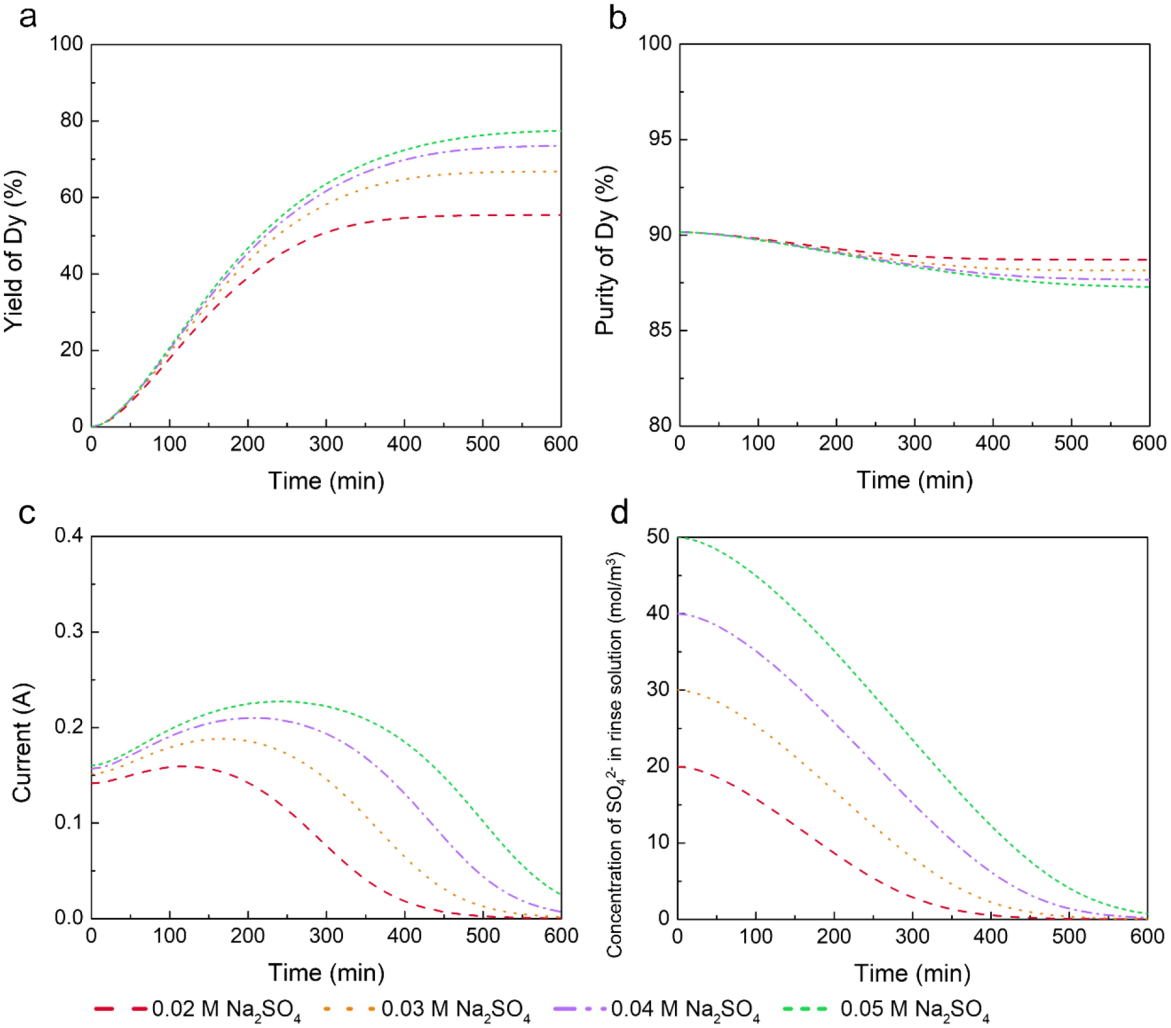
The separation performance and current during the process for different rinse solution concentrations. (**a**) Yield of Dy. (**b**) Purity of Dy. (**c**) Current. (**d**) Concentration of SO_4_^2−^ in the rinse solution. Other conditions: feed solution pH of 4, an EDTA/Dy molar ratio of 1, voltage of 4 V, and feed containing 1 mmol/L Pr(III), Nd(III), and Dy(III) at 25 °C.

Figure 5a,b display the yield and purity of Dy, respectively. It was observed that lower Na_2_SO_4_ concentrations resulted in a reduced Dy yield, with the process reaching a plateau earlier than with higher concentrations. This earlier plateau, indicating an earlier cessation of electrodialysis, is attributed to the depletion of SO_4_^2−^ in the rinse solution. As shown in Fig. 5c,d, this depletion leads to a cessation of current, effectively ending the ion migration and the electrodialysis process. The early depletion of SO_4_^2−^ hinders the complete removal of REEY^−^ from the feed compartment, leading to suboptimal separation performance.

To illustrate the trade-off effect of rinse solution concentration, Table 2 compares separation performance and energy efficiency across different Na_2_SO_4_ concentrations. With 0.02, 0.03, 0.04, and 0.05 mol/L Na_2_SO_4_, the final Dy yields achieved were 68%, 75%, 80%, and 82%, respectively, at varying times. Notably, the time efficiency substantially decreases with more dilute rinse solutions; for instance, in the 0.02 mol/L case, it took 276 min to increase the yield from 68.0 to 68.3%. Consequently, the time points for comparing the data in Table 2 were chosen when the electrodialysis process achieved 68%, 75%, 80%, and 82% Dy yield for each respective case.

**Table 2:** Electrodialysis time, Dy yield, purity, separation factor, and specific energy consumption at different rinse solution concentrations.

Rinse solution concentration (mol/L)	Electrodialysis time (min)	Dy yield (%)	Dy purity (%)	Separation factor	SEC (kWh/mol)
0.02	708	68	87	34	8
0.03	714	75	87	42	10
0.04	902	80	86	51	12
0.05	1032	82	86	56	14

While the yields of Dy varied, its purity remained consistent across different concentrations of the rinse solution, suggesting that reducing the rinse solution concentration has a negligible effect on Dy purity. However, specific energy consumption saw a significant reduction, decreasing from 14 kWh/mol at a concentration of 0.05 mol/L to 8 kWh/mol at 0.02 mol/L. This demonstrates a trade-off between yield and energy efficiency when lowering rinse solution concentration. Figure 4b illustrates this trade-off: initially, the flux of REE-chelate increases but subsequently declines sharply as more sulfate ions migrate into the feed compartment. In the latter stages, despite the ongoing migration of REE-chelates to the concentrate compartment, the process becomes less efficient due to the high flux of sulfate ions. The disproportionate flux of SO_4_^2−^ relative to REE-chelates reduces energy efficiency, and this negative effect is exacerbated with higher concentrations of SO_4_^2−^. To address this trade-off, implementing a continuous process could be beneficial. A continuous system, optimized to maintain a constant and favorable ratio of REE-chelate to SO_4_^2−^ flux, could enhance energy efficiency and mitigate the negative impacts observed in the batch process. This approach would optimize both the separation efficiency and the overall energy consumption of the process.

### Effect of feed solution concentration

In the separation stage of electrodialysis for REEs, the initial feed concentration is significantly influenced by the upstream extraction process. The concentration of total REEs varies depending on the source of the leachate. For instance, leachates from ionic clays typically exhibit very dilute concentrations of REEs, with one reported case below 500 mg/L^14,16^. In contrast, leachates from rock-forming ores such as monazites and bastnasites tend to have much higher REE concentrations up to a few g/L of REEs^17^. The feed concentration is a critical factor for process engineers to consider, as it should be optimized to achieve the best separation performance in terms of time–space yield and energy efficiency. Sometimes, processes like dilution or concentration are conducted to adjust the feed to a desirable concentration level before separation.

We simulated a broad range of initial feed concentrations, from 1 (447.7 mg/L) to 0.05 mol/m^3^ (22.9 g/L), to investigate the impact of initial concentration on separation efficiency. The lower end of this range approximates the concentration in leachates from ionic clays, while the upper end, although high, does not exceed the solubility limits of the REEs. The solubility of Pr_2_(SO_4_)_3_, Nd_2_(SO_4_)_3_, and Dy_2_(SO_4_)_3_ at 25 °C and a pH are reported to be 115, 51, and 36 g/L, respectively^1,18^. The separation conditions for these simulations were set at 8 V, the average value between the lowest and highest voltage values in this study, with a feed pH of 4 and an EDTA/Dy molar ratio of 1.

As mentioned in the previous section, there should be enough Na_2_SO_4_ in the rinse solution to avoid earlier cessation and insufficient separation of Dy. For each initial feed concentration in our electrodialysis study, we determined the corresponding amount of rinse solution concentration needed to achieve a consistent separation performance. Specifically, we aimed for a 82% yield and 86% purity of Dy upon completion. The required rinse solution concentrations were found to have a linear relationship with the feed conentration.

**Figure 6:**
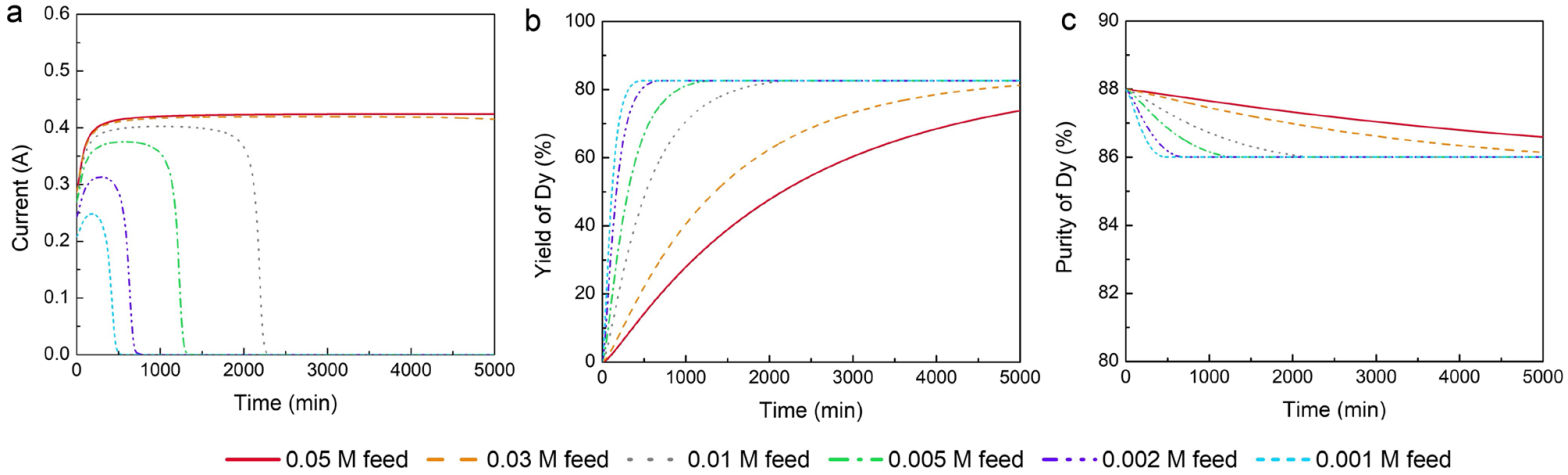
The performance of electrodialysis at different initial feed concentrations. (**a**) Current. (**b**) Yield of Dy. (**c**) Purity of Dy. other conditions: feed solution pH of 4, an EDTA/Dy molar ratio of 1, voltage of 8 V at 25 °C.

As both the feed and rinse solution concentrations increase, the system's conductivity also increases, resulting in higher current during the electrodialysis process. It is important to note that the Na_2_SO_4_ concentration required for higher concentration feeds is still within its solubility limits, making the separation process theoretically feasible. Figure 6a shows that the maximum current is around 0.45 A, which is still in the normal range of electrodialysis.

The yield and purity of Dy for different initial feed concentrations are presented in Fig. 6b,c, respectively. It was observed that different initial feed concentrations ultimately achieve the same separation performance in terms of Dy yield, purity, and separation factor, primarily because all feeds maintained the same EDTA/Dy ratio and pH level. As anticipated, increasing the feed concentration leads to longer separation times. However, upon comparing the rate of time increase to the rate of concentration increase, a noteworthy discovery emerges. A 49-fold increase in feed concentration results in only a 22-fold increase in separation time, suggesting that higher feed concentrations can enhance time efficiency.

To evaluate the energy efficiency of the electrodialysis process across different initial feed concentrations, we calculated specific energy consumption for each feed concentration. These calculations were made at the point where the yield of Dy was 82% and the purity was 86%.

The comparison results, as detailed in Table 3, reveal an interesting trend: although higher feed concentrations result in larger currents, the overall specific energy consumption decreases, ranging from 29 kWh/mol at the lowest concentration to 25 kWh/mol at the highest. Notably, the highest feed concentration of 0.05 mol/L REEs exhibits the best energy efficiency at 25 kWh/mol.

**Table 3:** Electrodialysis time, separation factor, and specific energy consumption at different initial feed concentrations.

Feed concentration of each REE (III) (mol/L)	Electrodialysis time (min)	Separation factor	SEC (kWh/mol)
0.001	387	56	29
0.002	575	56	28
0.005	1114	56	26
0.01	1988	56	25
0.03	5472	56	25
0.05	8923	56	24

Dy yield: 82%; Dy purity: 86%

This outcome indicates that operating the electrodialysis process with a 0.05 mol/L feed concentration cannot improve Dy yield and purity, but enhances both time and energy efficiency compared with operations with a lower 1 mmol/L feed concentration. These findings are significant because they demonstrate that higher initial feed concentrations can lead to more efficient use of energy and time, which is a crucial factor in the scalability and economic viability of the electrodialysis process for REE separation in industrial applications.

## Conclusion

This study successfully develops a model to simulate the separation of Dy from Pr and Nd in an electrodialysis system with two anion exchange membranes. The model addresses three critical aspects: equilibrium in the feed, ion exchange dynamics in the membrane, and ion flux during electrodialysis, validated by Chi-squared (χ^2^) testing and RMSE calculations, confirming its accuracy.

Key factors such as applied voltage, rinse solution concentration, and feed concentration were examined for their effects on separation performance and energy efficiency. Findings indicate that while higher voltages speed up separation, they reduce energy efficiency, making lower voltages more energy efficient. The study highlights the importance of rinse solution concentration in both performance and energy efficiency, noting that reducing it improves efficiency, but too dilute solutions can disrupt the process. Finally, higher feed concentrations improve specific energy consumption and dictate the necessary rinse concentration for effective separation, suggesting a balance is crucial for optimal results.

Regarding the electrodialysis cell configuration, the setup featuring two adjacent anion exchange membranes presents a challenge in terms of energy efficiency. This is primarily due to the independent migration of SO_4_^2−^ ions, which results in suboptimal energy efficiency compared with configurations that alternate between cation and anion exchange membranes. The independent migration of SO_4_^2−^ ions in this configuration leads to a disproportionate consumption of energy, hindering the overall efficiency of the process. Given this limitation, it is prudent to explore and investigate alternative cell configurations, particularly those that utilize a combination of cation and anion exchange membranes. Such configurations may offer improved energy efficiency by facilitating more balanced ion migration and reducing unnecessary energy expenditure.

Our future research will concentrate on the experimental investigation and optimization of these alternate cell configurations. We aim to conduct comprehensive studies to understand the dynamics of ion migration and energy consumption in these setups better. The goal is to identify and refine operational conditions that maximize separation efficiency while minimizing energy usage. This will involve exploring various membrane arrangements, voltage settings, rinse solution concentrations, and feed compositions to determine the most effective and efficient configuration for electrodialysis in REE separation. Through these efforts, we hope to contribute to the development of more sustainable and cost-effective methods for REE extraction and purification.

## Methodology

### Dissociation and chelation equilibrium in feed solution

The EDTA, represented as H_4_Y, is utilized as a chelating agent to enhance the selectivity of the separation process. It is capable of dissociating into five different species. The fully dissociated form, denoted as ${Y}^{4-}$, has the ability to form stable chelates with ${REE}^{3+}$ ions, resulting in the formation of ${REEY}^{-}$ chelates with a stable octahedral structure. The dissociation reactions of EDTA are outlined as follows^11^.

1 $$H_{4} Y \rightleftharpoons H_{3} Y^{ - } + H^{ + } \,\,\,\,\,\,\,K_{1} = \frac{{C_{{H_{3} Y^{ - } }} C_{{H^{ + } }} }}{{C_{{H_{4} Y}} }}$$

2 $${H}_{3}{Y}^{-}\rightleftharpoons {H}_{2}{Y}^{2-}+{H}^{+} \,\,\,\,\,\,\,\,{K}_{2}=\frac{{C}_{{H}_{2}{Y}^{2-}}{C}_{{H}^{+}}}{{C}_{{H}_{3}{Y}^{-}}}$$

3 $${H}_{2}{Y}^{2-}\rightleftharpoons H{Y}^{3-}+{H}^{+} \,\,\,\,\,\,\,{K}_{3}=\frac{{C}_{H{Y}^{3-}}{C}_{{H}^{+}}}{{C}_{{H}_{2}{Y}^{2-}}}$$

4 $$H{Y}^{3-}\rightleftharpoons {Y}^{4-}+{H}^{+} \,\,\,\,\,\,\,{K}_{4}=\frac{{C}_{{Y}^{4-}}{C}_{{H}^{+}}}{{C}_{H{Y}^{3-}}}$$

5 $${C}_{{H}^{+}}= {10}^{-pH}$$

where ${K}_{i}$ is the stepwise dissociation constant of EDTA, with the unit of mol L^−1^ (pK_1_ = 2.00, pK_2_ = 2.67, pK_3_ = 6.16, and pK_4_ = 10.26), and C_i_ is the concentration of species in the solution. The ${C}_{{H}^{+}}$ is the concentration of H^+^ calculated using Eq. (5) and feed pH.

When EDTA is introduced into the feed solution, chelation reactions occur. The chelation reaction between the Y^4−^ form of EDTA and ${REE}^{3+}$ ions, which include ${Pr}^{3+}$, ${Nd}^{3+}$, and ${Dy}^{3+}$, is represented as follows:

6 $${Y}^{4-}+{Pr}^{3+}\rightleftharpoons Pr{Y}^{-} \,\,\,\,\,\,\,{K}_{ABS,Pr}=\frac{{C}_{{PrY}^{-}}}{{C}_{{Y}^{4- }} {C}_{{Pr}^{3+}}}$$

7 $${Y}^{4-}+{Nd}^{3+}\rightleftharpoons Nd{Y}^{-} \,\,\,\,\,\,\,{K}_{ABS,Nd}=\frac{{C}_{{NdY}^{-}}}{{C}_{{Y}^{4- }} {C}_{{Nd}^{3+}}}$$

8 $${Y}^{4-}+{Dy}^{3+}\rightleftharpoons Dy{Y}^{-} \,\,\,\,\,\,\,{K}_{ABS,Dy}=\frac{{C}_{{DyY}^{-}}}{{C}_{{Y}^{4- }} {C}_{{Dy}^{3+}}}$$

In these chelation reactions, the absolute stability constant of the REEY^−^ complex, denoted as K_ABS_ (measured in mol L^−1^), plays a critical role. These constants reflect the affinity of the EDTA for the specific REE. The *pK*_*ABS*_ for Pr(III), Nd(III), and Dy(III) are 16.40, 16.61, and 18.30, respectively^11^.

When the feed solution in the electrodialysis process reaches equilibrium, it is necessary to calculate the concentrations of eleven different species including ${Pr}^{3+}, {Nd}^{3+}, {Dy}^{3+}, {PrY}^{-}, {NdY}^{-}, {DyY}^{-}, {H}_{4}Y,{H}_{3}{Y}^{-},{H}_{2}{Y}^{2-}, H{Y}^{3-}, and {Y}^{4-}.$ In order to determine the equilibrium concentrations of these eleven species, a set of eleven equations is required. This set includes the previously mentioned reactions in Eqs. (1)–(4) and Eqs. (6)–(8). Additionally, to fully solve for the equilibrium states, four mass balance equations are formulated. These equations are essential for accurately calculating the concentrations of each species in the solution at equilibrium, as demonstrated below:

9 $${C}_{Pr\_total}{=C}_{Pr{Y}^{-}}+{C}_{{Pr}^{3+}}$$

10 $${C}_{Nd\_total}={C}_{Nd{Y}^{-}}+{C}_{{Nd}^{3+}}$$

11 $${C}_{Dy\_total}{=C}_{Dy{Y}^{-}}+{C}_{{Dy}^{3+}}$$

12 $${C}_{EDTA\_total}=\sum_{i=1}^{3}{C}_{{REE}_{i}{Y}^{-}}+{{C}_{{H}_{4}Y}+C}_{{H}_{3}{Y}^{-}} {+C}_{{H}_{2}{Y}^{2-}} {+{C}_{H{Y}^{3-}}+ C}_{{Y}^{4-}}$$

Equations (9)–(11) represent the mass balance equations for each of the REEs, and Eq. (12) is the mass balance equation for EDTA. By simultaneously solving Eqs. (1)–(4) and Eqs. (6)–(12), it becomes possible to determine the concentrations of the eleven species in the feed solution. This comprehensive approach ensures an accurate calculation of each species' concentration within the system at equilibrium.

### Ion exchange equilibrium between the membrane and solution

When the feed solution is introduced into the feed compartment, anions present in the solution adsorb onto the anion exchange membrane (PC-400D) before they transition to the concentrate compartment. To determine the concentration of these anions within the membrane, the following equation, as detailed in the work of Crittenden et al.^19^, is used:

13 $$\overline{{q }_{i}}=\frac{\overline{{ q }_{T}}{\alpha }_{p}^{i}{q}_{i}}{{\sum }_{k=1}^{i}{\alpha }_{p}^{k}{q}_{k}}$$

where $\overline{{q }_{T}}$ (eq L^−1^) represents the adsorption capacity of the membrane. For the PC-400D membrane, this is quantified as 0.3714 eq L^−1^ (Supplementary Note S1). The selective factor for anion i, represented as ${\alpha }_{p}^{i}$, quantitatively describes the preference of anion i over the presaturant anion p on the adsorbent. Since the PC-400D membrane is typically stored in a NaCl solution, the presaturant ion in this case is Cl^−^. The term $\overline{{q }_{i}}$ (eq L^−1^) refers to the equivalent charge concentration of anion i within the membrane, while ${q}_{i}$ (eq L^−1^) is its equivalent charge concentration in the solution. It is important to note that the units used for adsorption equilibrium are based on equivalent charge concentration. However, the units in the feed equilibrium calculations are based on molarity. Consequently, a unit conversion is necessary for accurate calculations. For an ion with charge z and a concentration of c mol/L in the solution, the equivalent charge concentration in the solution would be $z\times c$ (eq L^−1^). This conversion is essential to align the measurements and ensure the precision of the calculations.

The schematic diagram in Fig. 1b suggests the migration of primary anions, $Pr{Y}^{-}$, $Nd{Y}^{-}$, $Dy{Y}^{-}$, and ${SO}_{4}^{2-}$ through the PC-400D membrane. These anions compete for adsorption on the membrane, eventually reaching an equilibrium. Their selectivity by the membrane is determined by the selective factor, ${\alpha }_{p}^{i}$, which largely depends on the anion's charge and molecular size. Typically, anions with larger charge values exhibit better adsorption. For ${SO}_{4}^{2-}$, the selective factor is known to be 9.1 for commercial anion-exchange materials, as reported by a previous study^19^. However, there is no established value for the selective factor of $REE{Y}^{-}$ in anion exchange membranes. Given the design of the PC-400D membrane, which is tailored for larger molecules, the model makes the following assumptions: the selective factors for $Pr{Y}^{-}$, $Nd{Y}^{-}$, $Dy{Y}^{-}$ are assumed to be 5, 7, and 9, respectively. These assumptions were refined and determined through model calibration.

Based on the ion concentrations in the feed compartment and Eq. (13), the concentrations of $Pr{Y}^{-}$, $Nd{Y}^{-}$, $Dy{Y}^{-}$, and ${SO}_{4}^{2-}$ within the membrane can be calculated. This approach allows for a more accurate prediction of ion behavior and efficiency of the electrodialysis process using the PC-400D membrane.

### Electrode reactions and overpotential

In electrodialysis, the hydrogen evolution reaction occurs at the cathode and the oxygen evolution reaction at the anode, both contributing to potential drops at their respective electrodes. The half-reactions and their electrode potentials are defined in Eqs. (14) and (15), based on conditions in neutral media^20^. The inherent potential drop from these electrode reactions is typically 1.23 V, as determined thermodynamically. However, the actual potential difference required to initiate these reactions is usually greater than 1.23 V, meaning the cathode potential exceeds + 0.423 V, and the anode potential is less than + 0.817 V. Overpotential, influenced by current density, is calculated using the Tafel equation—a simplified form of the Butler-Volmer equation when the absolute overpotential exceeds 0.1 V. This equation, presented as Eq. (16), is applied individually to calculate the overpotentials η^+^ at the cathode and η^−^ at the anode. The total overpotential is the aggregate of the absolute values of η^+^ and η^−^, as depicted in Eq. (17). The effective potential, outlined in Eq. (18), is then computed by subtracting 1.23 V and the total overpotential from the applied potential.

14 $$\text{Cathode}: {4H}_{2}O+4{e}^{-}\rightleftharpoons 2{H}_{2}+4{OH}^{-} \,\,\,\,\,\,\,{E}^{^\circ }=+0.423V$$

15 $$\text{Anode}:{2H}_{2}O\rightleftharpoons 4{e}^{-}+{O}_{2}+4{H}^{+} \,\,\,\,\,\,\,{E}^{^\circ }=+0.817V$$

16 $${\eta }_{\pm }= A\cdot log(\frac{i}{{i}_{0}})$$

17 $$\eta ={\eta }_{+}+{|\eta }_{-}|$$

18 $${U}_{e}=U-1.23 V-\eta$$

where ${E}^{^\circ }$ (V) is the electrode potential in the standard state, $\eta$, ${\eta }_{+}$, and ${\eta }_{-}$ are total overpotential (V), overpotential at the cathode, and overpotential at the anode, respectively. The A (V) is the Tafel slope and i (A m^−2^) is the current density. ${i}_{0}$ (A m^−2^) is the exchange current density that is determined by the electrode materials, ${U}_{e}$ is the effective potential (voltage, V) and U (V) is the applied potential.

The Tafel slope and exchange current density parameters used in the modeling are sourced from literature studies involving similar electrode materials. For the cathode made of stainless steel, the Tafel slope is set at − 0.14 V and the exchange current density at 0.015 A m^−2^^21^. For the anode composed of Ti/Pt material, the Tafel slope is 0.119 V with an exchange current density of 0.186 A m^−2^^22^.

During electrodialysis, the current density typically peaks and then eventually falls to zero. Consequently, the overpotential is estimated using half the value of the peak current density. Table 4 details the total overpotentials assumed, calculated based on varying peak current densities. If the actual current density falls outside the expected range, an adjusted overpotential is applied to match the observed conditions.

**Table 4:** Assumed overpotentials for different current ranges.

Current density range (A/m^2^)	Total overpotential (V)
0–10	0.601
0–20	0.679
0–40	0.757
0–60	0.803

### Ion migration through membranes

Upon the application of an electric potential, ion migration commences within the electrodialysis cell. As illustrated in Fig. 1b, separation occurs as the anions ($Pr{Y}^{-}$, $Nd{Y}^{-}$, $Dy{Y}^{-}$) in the feed compartment migrate through the PC-400D membrane, while the cations (${Pr}^{3+}$, ${Nd}^{3+}$, ${Dy}^{3+}$) are retained in the feed compartment. To effectively model this separation process, the focus is primarily on the migration of anions through the PC-400D membrane, as this is the critical step for achieving separation.

The migration of ions within the membrane, crucial for the separation, is described by the expanded Nernst-Planck equation. This equation is key to understanding and quantifying how ions move in response to the electric potential, and it is represented in Eq. (19). This approach allows for a detailed analysis of the ion transport dynamics in the membrane, which is essential for optimizing the electrodialysis separation process.

19 $$\overline{{J_{i} }} = - \overline{{D_{\iota } }} \nabla \overline{{C_{\iota } }} - \frac{{z_{i} \overline{{D_{\iota } }} \overline{{C_{\iota } }} F}}{RT}\nabla \emptyset + \overline{{C_{\iota } }} \nu$$

where $\overline{{J }_{i}}$, $\overline{{D }_{i}}$ and $\overline{{C }_{i}}$ are the flux (mol m^−2^ s^−1^), diffusion coefficient (m^2^ s^−1^) and concentration of ion i (mol m^−3^) in the membrane. The $\nabla \emptyset$ (V m^−1^) is the potential gradient of the membrane, and $\nu$ (m s^−1^) is the velocity of the solution in the compartment. The presence of steric hindrance in the membrane leads to a reduced diffusion coefficient compared with that in the solution. This reduction can vary significantly, typically ranging from 1/50 to 1/1000 of the value in the solution, as noted by Takahashi et al.^2^. In our model, it is assumed that the diffusion coefficient of $REE{Y}^{-}$ within the membrane is approximately 1/400 of its value in the solution. It is also assumed that the diffusion coefficient of SO_4_^2−^ in the membrane is 1/800 of its value in the solution due to its higher charge density. This assumption is based on the understanding of how steric hindrance affects ion mobility in membrane environments. The diffusion coefficients for various species in the solution, which are crucial for accurate modeling, are provided in Table 5.

**Table 5:** Diffusion coefficient of various ions in the solution^2,23,24^.

Species	Diffusion coefficient (m^2^ s^−1^)
Na^+^	$1.33\times {10}^{-9}$
Pr^3+^	$6.18\times {10}^{-10}$
Nd^3+^	$6.16\times {10}^{-10}$
Dy^3+^	$5.82\times {10}^{-10}$
SO_4_^2−^	$1.07\times {10}^{-9}$
$Pr{Y}^{-}$	$5.49\times {10}^{-10}$
$Nd{Y}^{-}$	$5.43\times {10}^{-10}$
$Dy{Y}^{-}$	$5.76\times {10}^{-10}$

*The diffusion coefficients cited from the literature were measured in dilute solutions, and we have assumed that the variance among these coefficients across different dilute solutions is negligible.

In the electrodialysis process, as described by the expanded Nernst-Planck equations, the total flux of an ion i comprises three distinct components: diffusion flux, electromigration flux, and convection flux^12^. It is important to note that the effective voltage in electrodialysis is usually lower than the applied voltage. This is due to the minimum thermodynamic energy necessary to initiate redox reactions at the electrodes^25^. While the theoretical minimum potential drop at the electrode is approximately 1.23 V, in practical situations, this value is typically higher^25^. This phenomenon was thoroughly examined in the preceding section, where the overpotential was determined based on the range of current density observed. The specific overpotential values used for each simulation case are detailed in Table S1 within the Supplementary Material.

The potential gradient for the electromigration flux in Eq. (19) can be nullified by applying the condition of electroneutrality in the membrane, as outlined in Eq. (20), combined with the condition of electric current flow described in Eq. (21). As a result, the ion flux can be effectively represented by Eq. (22). Also, the transport number of each ion can be calculated using Eq. (23). This approach allows for a more accurate calculation of ion movement, taking into account the various forces and constraints acting on ions in the electrodialysis process.

20 $$P=\sum_{i=1}^{n}\overline{{C }_{REEYi}}+2\times \overline{{C }_{{SO}_{4}^{2-}}}$$

21 $$i=F\sum_{i=1}^{n+2}{(z}_{i} \overline{{J }_{i}})$$

22 $$\overline{{J }_{i}}=-\overline{{D }_{i}}\frac{d\overline{{C }_{i}}}{dx}+\frac{{z}_{i}\overline{{D }_{i}} \overline{{C }_{i}}}{\sum_{i=1}^{4}{{z}_{i}}^{2}\overline{{D }_{i}} \overline{{C }_{i}}}(\frac{\text{i}}{F}+\sum_{i=1}^{4}{z}_{i}\overline{{D }_{i}} \frac{d\overline{{C }_{i}}}{dx})$$

23 $${t}_{i}=\frac{{z}_{i}\overline{{J }_{i}}}{\sum_{i=1}^{n+2}{z}_{i} \overline{{J }_{i}}}$$

where P (mol m^−3^) is the capacity of the PC-400D and $\overline{{C }_{i}}$ (mol m^−3^) is the concentration of anions in the membrane. The term i (A m^−2^) is the current density, F is the Faraday constant (96,486 C mol^−1^), ${z}_{i}$ is the ion charge, *x* (m) is the thickness of the membrane, and t_i_ is the transport number of the ions.

Given the assumption that the influences of diffusion and convection are negligible in comparison with the effect of the electronic current, Eq. (22) can be simplified. This simplification leads to Eq. (24), which primarily focuses on the impact of the electronic current in the electrodialysis process. This assumption allows for a more streamlined and focused analysis of ion flux, primarily driven by electromigration, which is often the dominant mechanism in many electrodialysis scenarios.

24 $$\overline{{J }_{i}}=\frac{{z}_{i}\overline{{D }_{i}} \overline{{C }_{i}}}{\sum_{i=1}^{4}{{z}_{i}}^{2}\overline{{D }_{i}} \overline{{C }_{i}}}\frac{\text{i}}{F}$$

In accordance with the principle of continuity, the ion flux in the solution is equal to the ion flux in the membrane. This means that the movement of ions within the solution and their transition through the membrane are consistent in terms of quantity. Equation (25) is formulated to define the ion flux in the solution. This equation ensures that the ion flux within the solution aligns with the ion flux occurring in the membrane, maintaining a balance and continuity in the ion transport process.

25 $${J}_{i}=\frac{V}{S}\frac{d{C}_{fi}}{dt}=-\frac{{z}_{i}\overline{{D }_{i}} \overline{{C }_{i}}}{\sum_{i=1}^{4}{{z}_{i}}^{2}\overline{{D }_{i}} \overline{{C }_{i}}}\frac{\text{i}}{F}$$

where ${J}_{i}$ is the ion flux in the solution (mol m^−2^ s^−1^), V (m^3^) is the volume of feed solution and S (m^2^) is the effective surface area for the membrane.

The ordinary differential equations (ODE) are obtained by rearranging Eq. (25) as follows:

26 $$\frac{d{C}_{fi}}{dt}=-\frac{S}{V}\frac{{z}_{i}\overline{{D }_{i}} \overline{{C }_{i}}}{\sum_{i=1}^{4}{{z}_{i}}^{2}\overline{{D }_{i}} \overline{{C }_{i}}} \frac{i}{F}$$

In the experimental phase of the study^11^, a noticeable time lag was observed between the onset of the experiment and the detection of ions in the concentrate compartment. This observation suggests that ions initially encounter resistance within the membrane. To account for this initial time lag, a mathematical expression $\frac{t+c}{t}$ is introduced into the ODEs governing the system. This expression is a fractional equation, where t appears in both the numerator and denominator, and c is a constant representing the duration of the time lag. The value of c is indicative of the significance of this lag at the beginning of the process; a larger value for c signifies a more pronounced initial time delay. As time t progresses, the value of the expression approaches 1, indicating that the ion flux increasingly conforms to the Nernst-Planck theory and reaches a quasi-steady state. In this state, the influence of the initial time lag diminishes. The primary function of this expression is to adjust the early part of the curve to reflect the observed time lag. Its impact on the main process is minimal. The constant c was determined and fine-tuned during the optimization stage of the model. This adjustment ensures that the model more accurately reflects the experimental observations, particularly during the initial stages of the electrodialysis process.

By expanding Eq. (26) for each ion and considering time lag, the following series of ODEs and three initial conditions are obtained:

27 $$\frac{d{C}_{f{PrY}^{-}}}{dt}=-\frac{S}{V}\frac{{z}_{{PrY}^{-}}\overline{{D }_{{PrY}^{-}}} \,\,\,\overline{{C }_{{PrY}^{-}}}}{\sum_{i=1}^{4}{{z}_{i}}^{2}\overline{{D }_{{REEY}_{i}^{-}}} \,\,\,\overline{{C }_{{REEY}_{i}^{-}}}}\frac{\text{i}}{F (\frac{t+c}{t})}$$

28 $$\frac{d{C}_{f{NdY}^{-}}}{dt}=-\frac{S}{V}\frac{{z}_{{NdY}^{-}}\overline{{D }_{{NdY}^{-}}} \,\,\,\overline{{C }_{{NdY}^{-}}}}{\sum_{i=1}^{4}{{z}_{i}}^{2}\overline{{D }_{{REEY}_{i}^{-}}} \,\,\,\overline{{C }_{{REEY}_{i}^{-}}}}\frac{\text{i}}{F (\frac{t+c}{t})}$$

29 $$\frac{d{C}_{f{DyY}^{-}}}{dt}=-\frac{S}{V}\frac{{z}_{{DyY}^{-}}\overline{{D }_{{DyY}^{-}}} \,\,\,\overline{{C }_{{DyY}^{-}}}}{\sum_{i=1}^{4}{{z}_{i}}^{2}\overline{{D }_{{REEY}_{i}^{-}}} \,\,\,\overline{{C }_{{REEY}_{i}^{-}}}}\frac{\text{i}}{F (\frac{t+c}{t})}$$

Initial conditions:

At t = 0

$${C}_{{PrY}^{-}}={C}_{{PrY}_{0}^{-}}$$

$${C}_{{NdY}^{-}}={C}_{{NdY}_{0}^{-}}$$

$${C}_{{DyY}^{-}}={C}_{{DyY}_{0}^{-}}$$

In the model's ordinary differential equations (ODEs), the concentration of ions within the membrane, denoted as $\overline{C }$, is determined by calculating the ion exchange equilibrium between the membrane and the solution. This concentration is then converted from eq L^−1^ to mol m^−3^ to ensure consistency in the units used throughout the model.

The terms z, S, V, F, and $\overline{D }$ are treated as constant values within the model. These constants play crucial roles in the mathematical representation of ion transport and flux. To solve the ODEs effectively, it is essential to express the current density i as a function of the ion concentration ${C}_{i}$. This relationship is critical for understanding how the concentration of ions in the solution influences the current density, and ultimately, the electrodialysis process. The specific formulation of this relationship and its application in the model will be detailed in the following section.

### Calculation of system resistances and current density

The current density (i, A m^−2^) is calculated as follows:

30 $$i=\frac{I}{S}$$

where I (A) is the system current, and is calculated using Ohm’s law:

31 $$I=\frac{{U}_{e}}{{R}_{total}}$$

where ${U}_{e}$ is effective voltage in V, and R_total_ (Ω) is the total resistance of the system. The total resistance of the system consists of resistance of feed compartments (R_f_), concentrate compartments (R_c_), rinse compartments (R_r_) and all ion exchange membranes (R_AMX_, R_CMX_, and R_PC-400D_), as following:

32 $${R}_{total}={R}_{f}+{R}_{c}+{2\times R}_{r}+{R}_{CMX}+{R}_{AMX}+{R}_{PC-400D}$$

The resistances of membranes can be calculated using Eq. (33), where $\rho$ is the area resistance ($\Omega$ m^2^) obtained from manufacture. The S (m^2^) is the surface area of the membrane, and $\upbeta$ is the masked fraction of the membrane by the spacer.

The area resistance data provided by the manufacturer, based on the behavior of Na^+^ and Cl^−^ ions in high concentration environments such as 0.5 mol/L NaCl for CMX and AMX membranes, does not accurately reflect the conditions in our experiments which involve much more dilute solutions, approximately 0.001 mol/L. Furthermore, the larger molecular size of REE-chelates and SO_4_^2−^ ions creates significant steric hindrance, further impeding their movement through the membrane. Consequently, the actual membrane resistance in our setup is likely to be higher than the values predicted using Eq. (33).

To account for this discrepancy, a constant "b" was introduced into Eq. (33), leading to the modified Eq. (34). This constant "b" effectively adjusts the area resistance in the model to reflect the actual behavior of the larger ions in the system. Drawing on the relationship between solution concentration and area resistance outlined in the study by Galama et al.^26^, the value of b = 45 was selected and applied across all modeling and simulation scenarios in our study. This value was empirically tested and confirmed to be appropriate for representing the specific conditions of our experiments. This adjustment ensures that the model accurately reflects the higher resistance due to low solution concentrations and the steric hindrance of larger ions, thereby enhancing the reliability and predictive accuracy of the model.

33 $${R}_{Membrane}=\frac{\rho }{S(1-\beta )}$$

34 $${R}_{CMX} \,or \,{R}_{AMX} \,or \,{R}_{PC-400D}=\frac{\rho b}{S(1-\beta )}$$

The resistance of solution can be calculated from the solution specific conductivity, as shown in Eq. (35):

35 $${R}_{c} \,or \,{R}_{f} \,or \,{R}_{r} =\frac{l}{\kappa S{\varepsilon }^{2}}$$

where l (m) is the distance between membranes (compartment thickness or spacer width), $\kappa$ (S m^−1^) is the specific conductivity, S (m^2^) is the active area of the ion exchange membranes, and $\varepsilon$ is the porosity of the spacer (the porosity is squared to reflect the tortuous ion transport)^12^. The tortuosity ($\tau$) is the average ratio between the actual path length inside the porous medium to straight distance connecting the two points.

36 $$\tau =\frac{actual \,path \,length}{straight \,distance}$$

In the context of electrodialysis, the compartment is often treated as a porous medium, a characteristic attributed to the structure of the spacer within it. This structural aspect of the spacer means that the actual path taken by anions to the membrane is longer than the straightforward measurement of the compartment's thickness would suggest^27^.

37 $$\uptau =\frac{1}{{\varepsilon }^{2}}$$

The porosity of the spacer is typically specified by the manufacturer. In this study, we worked with a spacer having a porosity of 0.7. This specific porosity value leads to a tortuosity factor of 2.

The specific conductivity is determined by the mobility and concentration of cations and anions in the solution:

38 $$\kappa =\sum {n}_{i}{Q}_{i}{\mu }_{i}$$

where ${n}_{i}$ (m^−3^) is the number concentration of ion *i* (both cations and anions), ${Q}_{i}$ (C) is the charge of ion *i*, and ${\mu }_{i}$ (m^2^ V^−1^ s^−1^) is the ion mobility of the ion *i*. The ion mobility can be calculated from diffusion coefficient based on the Nernst Einstein equation:

39 $${\mu }_{i}=\frac{{z}_{i}e{D}_{i}}{{k}_{B}T}$$

where *z*_*i*_ is the charge of ion i, *e* is elementary charge (1.602 × 10^–19^ C), *D*_*i*_ is the diffusion coefficient of ion* i* (from Table 4), *k*_*B*_ is the Boltzmann constant (1.380649 × 10^–23^ m^2^ kg s^−2^ K^−1^) and T (K) is temperature.

The *n*_*i*_ and *Q*_*i*_ in Eq. (38) are calculated as follows:

40 $${n}_{i}={N}_{A}{C}_{i}$$

41 $${Q}_{i}={z}_{i}e$$

where N_A_ is the Avogadro’s number (${N}_{A}= 6.022\times {10}^{23}$ mol^−1^) and C_i_ (mol m^−3^) is the concentration of ions in each compartment.

By resolving Eqs. (30)–(35), it is possible to express the current density (i) as a function of the ion concentration (C_i_) in the solution. This relationship is crucial in electrodialysis models as it links the electrical aspect of the process (current density) with the chemical composition of the solution (ion concentration).

### Solving the ordinary differential equations

In this study, the ODEs are formulated as functions of the concentrations of ${PrY}^{-}$, ${NdY}^{-}$, and ${DyY}^{-}$, i.e., ${C}_{fPr{Y}^{-}}$, ${C}_{fNd{Y}^{-}}$, and ${C}_{fDy{Y}^{-}}$, in the feed solution. To solve these ODEs, the Runge–Kutta 4th order method was employed, a numerical integration technique well-suited for dealing with such equations. In our approach, MATLAB functions were used to implement this method. The numerical integration process was conducted in a stepwise manner with a time increment of h = 60 s, allowing for a detailed and precise simulation of the changes in ion concentrations over time.

For the sake of simplicity in the mathematical formulation, we denoted ${C}_{fPr{Y}^{-}}$, ${C}_{fNd{Y}^{-}}$, and ${C}_{fDy{Y}^{-}}$ as y_1_, y_2_, and y_3_, respectively. This notation streamlines the representation of the equations and the corresponding calculations in MATLAB. By applying the Runge–Kutta 4th order method to these equations (Supplementary Note S2), we were able to numerically solve for the concentrations of the ${PrY}^{-}$, ${NdY}^{-}$, and ${DyY}^{-}$ chelates in the feed solution, thereby providing a detailed understanding of their behavior and dynamics within the electrodialysis process.

42 $$\begin{gathered} \frac{{dy_{1} }}{dt} = f_{1} \left( {y_{1} ,y_{2} ,y_{3} ,t} \right) \hfill \\ \frac{{dy_{2} }}{dt} = f_{2} \left( {y_{1} ,y_{2} ,y_{3} ,t} \right) \hfill \\ \frac{{dy_{3} }}{dt} = f_{3} \left( {y_{1} ,y_{2} ,y_{3} ,t} \right) \hfill \\ \end{gathered}$$

43 $$\begin{gathered} {\text{Initial conditions}}: \hfill \\ {\text{At t}} = 0 \hfill \\ y_{1} = y_{1,0} \hfill \\ y_{2} = y_{2,0} \hfill \\ y_{3} = y_{3,0} \hfill \\ \end{gathered}$$

### Model validation

To assess the accuracy of the model, we compared its predicted values against the actual experimental data. During the electrodialysis process, it was observed that the majority of Dy(III) ions migrated to the concentrate compartment, while most of the Pr(III) and Nd(III) ions remained in the feed compartment. Consequently, for the purpose of comparison between the model predictions and experimental results, we focused on the concentrations of Pr and Nd in the feed compartment and the concentration of Dy in the concentrate compartment.

To evaluate how well the model represents the experimental data, a Chi-squared (χ^2^) test was conducted, as outlined in Eq. (44). This statistical test is crucial for determining whether there is a significant difference between the expected model outcomes and the observed experimental results.

Additionally, the Root-Mean-Square Error (RMSE) was calculated, as shown in Eq. (45). The RMSE is used to quantify the average magnitude of the differences between values predicted by the model and the values actually observed in the experiments. The RMSE is a standard measure of the accuracy of a model, with lower values indicating a closer fit to the experimental data. Together, the χ^2^ test and RMSE provide a comprehensive evaluation of the model's fitness, offering insights into its reliability and accuracy in predicting the behavior of ions during the electrodialysis process.

44 $${\chi }^{2}=\sum \frac{{({O}_{i}-{E}_{i})}^{2}}{{E}_{i}}$$

45 $$RMSE= \sqrt{\frac{\sum {({O}_{i}-{E}_{i})}^{2} }{N}}$$

where O_i_ is the observed experimental data and E_i_ is the expected value calculated using the model. The N represents the number of paired observations.

### System key performance indicator (KPI) and energy efficiency

In this study, the model is employed to simulate experiments and explore various parameters, such as voltage, rinse solution concentration, and initial feed concentration. These simulations are aimed at estimating the performance and energy efficiency of the separation process.

For assessing the performance, several key performance indicators (KPIs) were calculated. These KPIs include the purity and yield of Dy, as well as the separation factor. These metrics are essential for evaluating the effectiveness of the separation process. The purity of Dy is calculated using Eq. (46). This equation helps quantify the proportion of Dy in the concentrate compartment relative to other ions, providing an indication of how effectively the process isolates Dy. The yield of Dy is determined using Eq. (47). This calculation measures the amount of Dy recovered in the concentrate compartment as a percentage of its initial amount in the feed, indicating the efficiency of the Dy recovery process. The separation factor is calculated using Eq. (48). This factor assesses the ability of the process to differentiate and separate Dy from other ions, particularly Pr and Nd, which is crucial for the effectiveness of the electrodialysis process.

46 $$Purity (\%)=\frac{{C}_{Dy,concentrate,final}}{{(\sum_{i=1}^{n}{C}_{i,total})}_{concentrate, final}}\times 100$$

47 $$Yield (\%)=\frac{{{(C}_{Dy, total})}_{concentrate,final}}{{{(C}_{Dy, total})}_{feed, initial}}\times 100$$

48 $$SF=\frac{\frac{{({C}_{Dy})}_{concentrate}}{{({C}_{Dy})}_{total,feed,final}}}{\frac{{({C}_{Nd(III)}+{C}_{\text{Pr}(III)})}_{concentrate}}{{({C}_{Nd(III)}+{C}_{\text{Pr}(III)})}_{total,feed,final}}}$$

To evaluate the energy efficiency of the Dy recovery process in the electrodialysis setup, the critical metric was calculated: specific energy consumption (SEC, Eq. (49)). The SEC metric is used to determine the amount of energy required to recover a specific amount of Dy. It is a key indicator of the process's energy efficiency, illustrating how much energy is expended to achieve a certain level of recovery. Lower values of SEC indicate a more energy-efficient process, which is especially important in scaling up for industrial applications where energy costs significantly impact overall feasibility and sustainability.

By analyzing these metrics, researchers and engineers can identify areas for improvement, optimize the process for energy use, and make it more viable for large-scale applications.

49 $$SEC= \frac{U{\int }_{0}^{t}Idt}{{n}_{i}}$$

where SEC (kWh mol^−1^) is the energy consumed for recovering 1 mol of Dy, U (V) is applied potential, I (A) is current, t (s) is operating time, and ${n}_{i}$ (mol) is the amount of recovered target ion (Dy).

### Supplementary Information

Supplementary Information.

## Data Availability

The data will be made available upon request. Please contact the corresponding author, Dr. Gisele Azimi (g.azimi@utoronto.ca).

## Abbreviations

A: Tafel slope (V)
$$C$$: Concentration (mol m^−^^3^)
$\overline{C }$: Concentration in the membrane (mol m^−^^3^)
$D$: Diffusion coefficient in the solution (m^2^ s^−^^1^)
$\overline{D }$: Diffusion coefficient in the membrane (m^2^ s^−^^1^)
$F$: Faraday constant (C mol^−^^1^)
$$I$$: Current (A)
$J$: Ion flux in the solution (mol m^−^^2^ s^−^^1^)
$\overline{J }$: Ion flux in the membrane (mol m^−^^2^ s^−^^1^)
$K$: Dissociation constant of EDTA (dm^3^ mol^−^^1^)
${K}_{ABS}$: Absolute stability constant (dm^3^ mol^−^^1^)
$KPI$: Key performance indicator
$N$: Number of pairs
${N}_{A}$: Avogadro’s number ($6.022\times {10}^{23}$ mol^−^^1^)
$ODE$: Ordinary differential equation
$P$: Membrane adsorption capacity (mol m^−^^3^)
$Q$: Charge of ion (C)
$R$: Gas constant (J mol^−^^1^ K^−^^1^)
$${R}_{i}$$: Resistance (Ω)
$REE$: Rare earth element
$RMSE$: Root-mean-square error
$S$: Surface area of the membrane (m^2^)
$SEC$: Specific energy consumption (KWh mol^−^^1^)
$$SF$$: Separation factor
$$T$$: Temperature (K)
*t*_*i*_: Transport number
$$U$$: Applied voltage (V)
$${U}_{e}$$: Effective voltage (V)
$V$: Volume of the solution (m^3^)
$b$: Constant parameter for membrane resistance
$c$: Constant parameter for time lag (s)
$$e$$: Elementary charge (1.602 × 10^–^^19^ C)
$h$: Time step in the model (s)
$$i$$: Current density (A m^−^^2^)
${i}_{0}$: Exchange current density (A m^−^^2^)
${k}_{B}$: Boltzmann constant (1.380649 × 10^–^^23^ m^2^ kg s^−^^2^ K^−^^1^)
$n$: Amount of the target ion I (m^−^^3^)
$\overline{q }$: Equivalent charge concentration in the membrane (eq dm^−^^3^)
$q$: Equivalent charge concentration in the solution (eq dm^−^^3^)
$$t$$: Time (s)
$x$: Thickness of the membrane (m)
$$z$$: Ion charge
$${\alpha }_{p}^{i}$$: Selective factor
$\beta$: Masked fraction of the membrane
$\varepsilon$: Porosity of the spacer
$$\eta$$: Total overpotential (V)
${\eta }_{-}$: Overpotential at the anode (V)
${\eta }_{+}$: Overpotential at the cathode (V)
$$\kappa$$: Conductivity (S m^−^^1^)
$$\mu$$: Ion mobility (m^2^ V^−^^1^ s^−^^1^)
$$\nu$$: Flow velocity (m s^−^^1^)
$$\tau$$: Tortuosity
$$\varnothing$$: Electric potential (V)
