# Supplementary material for: Mathematical modeling of rare earth element separation in electrodialysis with adjacent anion exchange membranes and ethylenediaminetetraacetic acid as chelating agent
Source: Sci Rep. 2024 May 28;14:12240. doi: 10.1038/s41598-024-62885-4 (PMC11133418; doi:10.1038/s41598-024-62885-4)
Supplement: Supplementary file 1 — Supplementary Information. [file 41598_2024_62885_MOESM1_ESM.docx]

**Supplementary Materials**

**Mathematical Modeling of Rare Earth Element Separation in Electrodialysis with Adjacent Anion Exchange Membranes and Ethylenediaminetetraacetic Acid as Chelating Agent**

Lingyang Ding^a^, Gisele Azimi ^a,^*

^a^Laboratory for Strategic Materials, Department of Chemical Engineering and Applied Chemistry, University of Toronto, 200 College Street, Toronto, Ontario M5S 3E5, Canada

*Corresponding author: [g.azimi@utoronto.ca](mailto:g.azimi@utoronto.ca)

Supporting Information Contents

3 Pages (including the cover page)

2 Notes

1 Table

**Note 1.** Adsorption capacity of the membrane

**Note 2.** Runge-Kutta 4^th^ order method

**Table S1.** List of varying constants used in the simulations.

**Note 1: Adsorption capacity of the membrane**

The ion exchange capacity of the PC-400D membrane, as specified by the manufacturer, is 0.00035 equivalents per gram on a dry basis. The thickness of the dry membrane varies between 0.016 cm and 0.02 cm, with an average value of 0.018 cm being utilized. A 10×10 cm^2^ section of the wet membrane was dried and weighed, totaling 1.910 g. Utilizing this data, the "adsorption capacity" of the membrane was determined through the calculations outlined below:

Adsorption capacity = $0.00035\frac{eq}{g}\times$ $\frac{1.910 g}{10cm\times10cm\times0.018cm}\times\frac{1000 {cm}^{3}}{1 L}=0.3714\frac{eq}{L}$ (1)

**Note 2: Runge-Kutta 4^th^ order method**

k_11_ = f_1_(y_1_, y_2_, y_3_)

k_21_ = f_2_(y_1_, y_2_, y_3_)

k_31_ = f_3_(y_1_, y_2_, y_3_)

k_12_ = f_1_(y_1_+0.5$\times$k_11_$\times$h, y_2_+0.5$\times$k_21_$\times$h, y_3_+0.5$\times$k_31_$\times$h)

k_22_ = f_2_(y_1_+0.5$\times$k_11_$\times$h, y_2_+0.5$\times$k_21_$\times$h, y_3_+0.5$\times$k_31_$\times$h)

k_32_ = f_3_(y_1_+0.5$\times$k_11_$\times$h, y_2_+0.5$\times$k_21_$\times$h, y_3_+0.5$\times$k_31_$\times$h)

k_13_ = f_1_(y_1_+0.5$\times$k_12_$\times$h, y_2_+0.5$\times$k_22_$\times$h, y_3_+0.5$\times$k_32_$\times$h)

k_23_ = f_2_(y_1_+0.5$\times$k_12_$\times$h, y_2_+0.5$\times$k_22_$\times$h, y_3_+0.5$\times$k_32_$\times$h)

k_33_ = f_3_(y_1_+0.5$\times$k_12_$\times$h, y_2_+0.5$\times$k_22_$\times$h, y_3_+0.5$\times$k_32_$\times$h)

k_14_ = f_1_(y_1_+k_13_$\times$h, y_2_+k_23_$\times$h, y_3_+k_33_$\times$h)

k_24_ = f_2_(y_1_+k_13_$\times$h, y_2_+k_23_$\times$h, y_3_+k_33_$\times$h)

k_34_ = f_3_(y_1_+k_13_$\times$h, y_2_+k_23_$\times$h, y_3_+k_33_$\times$h)

y_1_ = y_1_+(h/6)$\times$(k_11_+2$\times$k_12_+2$\times$k_13_+k_14_)

y_2_ = y_2_+(h/6)$\times$(k_21_+2$\times$k_22_+2$\times$k_23_+k_24_)

y_3_ = y_3_+(h/6)$\times$(k_31_+2$\times$k_32_+2$\times$k_33_+k_34_)

**Table S1.** List of varying constants used in the simulations.

| No. | Applied voltage (V) | Rinse solution concentration (mol/L) | Feed concentration (mol/L) | Overpotential η (V) | Time lag constant c (s) |
| --- | --- | --- | --- | --- | --- |
| 1 | 4 | 0.05 | 0.001 | 0.601 | 3000 |
| 2 | 6 | 0.05 | 0.001 | 0.679 | 2000 |
| 3 | 8 | 0.05 | 0.001 | 0.757 | 1500 |
| 4 | 10 | 0.05 | 0.001 | 0.757 | 1000 |
| 5 | 12 | 0.05 | 0.001 | 0.757 | 600 |
| 6 | 14 | 0.05 | 0.001 | 0.803 | 600 |
| 7 | 4 | 0.02 | 0.001 | 0.601 | 3000 |
| 8 | 4 | 0.03 | 0.001 | 0.601 | 3000 |
| 9 | 4 | 0.04 | 0.001 | 0.601 | 3000 |
| 10 | 8 | 0.1 | 0.002 | 0.757 | 1500 |
| 11 | 8 | 0.25 | 0.005 | 0.757 | 1500 |
| 12 | 8 | 0.5 | 0.01 | 0.757 | 1500 |
| 13 | 8 | 1.5 | 0.03 | 0.803 | 1500 |
| 14 | 8 | 2.5 | 0.05 | 0.803 | 1500 |
